# Diagnostic and Prognostic Value of Monocyte-to-Lymphocyte Ratio and Red Cell Distribution Width to Lymphocyte Ratio in Primary Biliary Cholangitis

**DOI:** 10.5152/tjg.2023.21768

**Published:** 2023-02-01

**Authors:** Yanting Jiang, Jian Wang, Huiling Wu, Wei Zhou, Sihui Li, Min Jin

**Affiliations:** Department of Clinical Laboratory, First Affiliated Hospital of Guangxi Medical University Nanning, Guangxi, China

**Keywords:** Diagnosis, monocyte-to-lymphocyte ratio, primary biliary cholangitis, prognosis, red cell distribution width-to-lymphocyte ratio

## Abstract

**Background:**

The aim of this study is to assess levels of monocyte-to-lymphocyte ratio and red cell distribution width-to-lymphocyte ratio in primary biliary cholangitis patients and excavate their clinical significance.

**Methods:**

The levels of monocyte-to-lymphocyte ratio and red cell distribution width-to-lymphocyte ratio in the primary biliary cholangitis, autoimmune hepatitis, and healthy controls were compared, and correlations between monocyte-to-lymphocyte ratio, red cell distribution width-to-lymphocyte ratio, and Mayo score were analyzed. The area under the receiver operating characteristic curve was utilized to analyze the diagnostic value of monocyte-to-lymphocyte ratio and red cell distribution width-to-lymphocyte ratio for primary biliary cholangitis.

**Results:**

Monocyte-to-lymphocyte ratio and red cell distribution width-to-lymphocyte ratio in primary biliary cholangitis were higher than they were in autoimmune hepatitis and healthy controls (each, *P* < .05). Area under the s of monocyte-to-lymphocyte ratio and red cell distribution width-to-lymphocyte ratio in diagnosis of primary biliary cholangitis were 0.821 and 0.797, respectively (each, *P* < .001). The combination of monocyte-to-lymphocyte ratio and red cell distribution width-to-lymphocyte ratio increased the diagnostic value of primary biliary cholangitis (area under the receiver operating characteristic curve = 0.868, *P* < .001). The correlation analysis showed that monocyte-to-lymphocyte ratio and red cell distribution width-to-lymphocyte ratio were positively correlated with Mayo score (r-MLR = 0.459, r-RLR = 0.522, *P* < .001 for each). Red cell distribution width-to-lymphocyte ratio was independently associated with Mayo score (*P* = .036) by multiple linear regression. In primary biliary cholangitis patients with Child–Pugh classification, monocyte-to-lymphocyte ratio and red cell distribution width-to-lymphocyte ratio levels in class B and class C were significantly higher than in class A (each, *P* < .05).

**Conclusion:**

Elevated levels of monocyte-to-lymphocyte ratio and red cell distribution width-to-lymphocyte ratio may prove to be useful markers for estimating the prognosis of primary biliary cholangitis, and the combined detection of monocyte-to-lymphocyte ratio and red cell distribution width-to-lymphocyte ratio has some clinical diagnostic value in patients with primary biliary cholangitis.

**Main Points**

- This article discusses the combined diagnostic value of monocyte-to-lymphocyte ratio and red cell distribution width-to-lymphocyte ratio in primary biliary cholangitis for the first time.
- This article shows that monocyte-to-lymphocyte ratio and red cell distribution width-to-lymphocyte ratio had good diagnostic value in patients with early primary biliary cholangitis, and the combined diagnostic value was even better.
- This article indicates that monocyte-to-lymphocyte ratio and red cell distribution width-to-lymphocyte ratio were positively correlated with the prognosis of primary biliary cholangitis.
- Red cell distribution width-to-lymphocyte ratio was an independent factor related to the prognosis of primary biliary cholangitis disease.
- Monocyte-to-lymphocyte ratio and red cell distribution width-to-lymphocyte ratio were higher in class B|C than in class A of the Child–Pugh classification.

## Introduction

Primary biliary cholangitis (PBC), also known as primary biliary cirrhosis, is an autoimmune liver disease that mainly occurs in adult women and is characterized by chronic immune-mediated destruction of small-sized and medium-sized bile ducts.^1^ Primary biliary cholangitis can be diagnosed if at least 2 of the following 3 criteria are met: (1) biochemical evidence of cholestasis based on alkaline phosphatase (ALP) elevation; (2) presence of antimitochondrial autoantibodies (AMA), or other PBC-specific auto antibodies, including sp100 or gp210, if AMA is negative; (3) histologic evidence of nonsuppurative destructive cholangitis and destruction of interlobular bile ducts.^2^

Although AMA can be present early in PBC patients, 5% to 10% of them are AMA negative,^3^ leading to misdiagnosis. In recent years, anti-sp100 and anti-gp210 antibodies are new biomarkers for the diagnosis of PBC, but they have relatively low sensitivity and are unable to timely assess the prognosis of disease.^4,5^ Currently, liver biopsy remains the gold standard for diagnosing and staging of PBC.^6^ However, because of the invasive nature of liver biopsy and the risk of complications, many patients are reluctant to undergo the procedure, omission of which can make a clinical diagnosis more difficult. Therefore, it is urgent to find new indicators to enable early clinical diagnosis and assessment of prognosis.

Primary biliary cholangitis is a chronic autoimmune disease, and the continuous inflammatory response is the key factor in its progression to liver fibrosis or cirrhosis.^7^ A recent study indicated that autoimmune liver disease is associated with an increase in novel inflammatory markers in the bloodstream.^8^ Monocyte-to-lymphocyte ratio (MLR) and red cell distribution width (RDW)-to-lymphocyte ratio (RLR) have been studied in a variety of inflammatory conditions. For instance, elevated MLR is associated with the severity and prognosis of cardiovascular disease, liver disease, and cancer.^9-11^ Red cell distribution width-to-lymphocyte ratio is a potential marker of inflammation. Recent studies have reported that increased RLR is significant in predicting fibrosis and cirrhosis in patients with chronic hepatitis.^12,13^

Although there has been extensive investigation of the association of MLR and RLR with many diseases, there are few studies about the correlation between MLR, RLR, and PBC. The purpose of the present study is to research the correlation between MLR, RLR, and disease prognosis in PBC patients and to excavate the clinical diagnostic value of these 2 indicators.

## MATERIALS AND METHODS

### Subjects

The clinical data of 71 patients (4 males and 67 females) diagnosed with PBC in the First Affiliated Hospital of Guangxi Medical University from January 2015 to May 2021 were retrospectively analyzed. The inclusion criterion was patients with liver biopsy-proven PBC. Exclusion criteria are (1) the presence of other liver diseases, such as autoimmune hepatitis (AIH), viral hepatitis, nonalcoholic fatty liver disease, liver cancer, and drug-induced liver injury; (2) complications caused by severe abnormalities of the cardiovascular, hematological, immune, or endocrine systems; and (3) coinfection. A total of 170 patients diagnosed with AIH at the same hospital during the same period were chosen as members of the disease control. It included 26 males and 144 females. All diagnoses of AIH met 2019 Practice Guidance and Guidelines from the American Association for the Study of Liver Diseases.^14^ In addition, 170 age- and gender-matched healthy volunteers who underwent a routine physical examination in the same hospital during the same period were randomly selected as healthy controls (HCs). The study was made in accordance with the Declaration of Helsinki and was subject to approval by the ethics committee of the First Affiliated Hospital of Guangxi Medical University (approval number: 2021 [K-Y-E-167]). All patients provided written informed consent.

### Data Extraction

Clinical characteristics and laboratory data were collected from patient medical records. In addition to age and gender, these data included levels of white blood cell count, hemoglobin, platelets, neutrophils, lymphocytes, monocytes, and RDW. Monocyte-to-lymphocyte ratio was calculated as the ratio of monocytes to lymphocytes, and RLR was calculated as the ratio of RDW to lymphocytes. Liver function indicators were gamma-glutamyl transferase (GGT), alanine aminotransferase (ALT), aspartate aminotransferase (AST), and ALP. Inflammatory markers were complement 3 (C3), complement 4 (C4), immunoglobulin A (IgA), immunoglobulin G (IgG), immunoglobulin M, and erythrocyte sedimentation rate.

### Mayo Score Model and Child–Pugh Classification Standard

The Mayo score model^15^ is used to predict the progression of PBC and to assess a prognosis. The Child–Pugh classification^16^ is a standard for quantitatively evaluating hepatic functional reserve. The classes A, B, and C indicate 3 different degrees of liver damage. Class A indicates compensated cirrhosis, and classes B and C (B|C) indicate decompensated cirrhosis.^17^

### Statistical Analysis

All statistical analyses were carried out using IBM SPSS Statistics version 25.0 (IBM Corp.; Armonk, NY, USA) and GraphPad Prism 8. The normally distributed quantitative data were expressed as means and standard deviations, and the non-normally distributed data were expressed as median (25th, 75th percentiles). One-way analysis of variance was utilized to compare the normal distribution data, and the Kruskal–Wallis test was utilized to compare the non-normally distributed data. The *χ* ^2^ test was utilized to compare gender. The Pearson correlation test or Spearman correlation test was utilized to perform correlation analysis of corresponding indexes. Receiver operating characteristic (ROC) curve analysis was carried out to determine the predictive value of PBC diagnosis. The result *P* < .05 indicated that the difference was statistically significant.

## Results

### Characteristics of Subjects

Seventy-one PBC, 170 AIH, and 170 HCs with laboratory test results are given in Table 1. There was no statistically significant difference in age and gender among PBC, AIH, and HCs. Red cell distribution width, MLR, RLR, GGT, and ALP were higher in PBC than in AIH and HCs (*P* < .001). Levels of hemoglobin, platelets, neutrophils, and lymphocytes were lower in PBC than in AIH and HCs (*P* < .001).

### Monocyte-to-lymphocyte ratio and Red Cell Distribution Width-to-Lymphocyte Ratio of Primary Biliary Cholangitis, Autoimmune Hepatitis, and Healthy Controls

**Figure 1.**
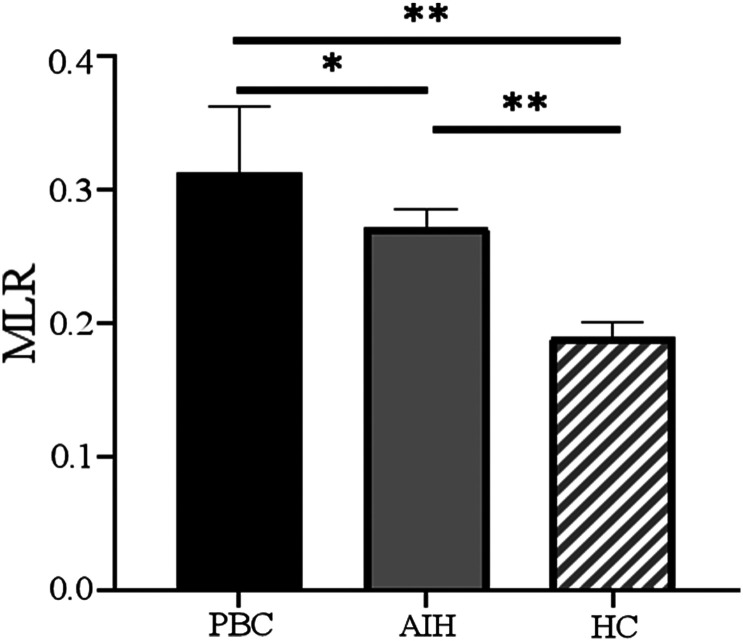
Comparison of MLR among PBC, autoimmune hepatitis, and healthy controls. *P < .05, **P < .01. MLR, monocyte-to-lymphocyte ratio; PBC, primary biliary cholangitis.

**Figure 2.**
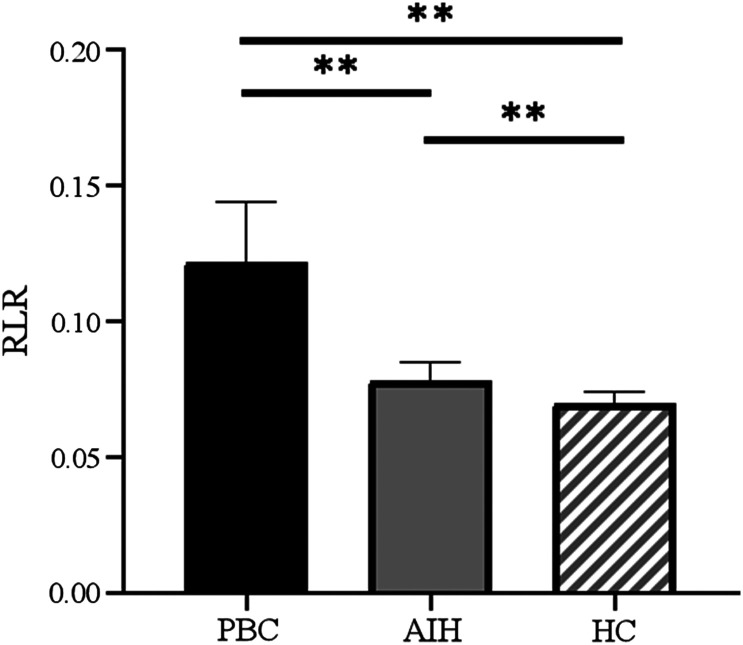
Comparison of RLR among PBC, autoimmune hepatitis, and healthy controls. *P < .05, **P < .01. RLR, red cell distribution width-to-lymphocyte ratio; PBC, primary biliary cholangitis.

The MLR of PBC was higher than that of AIH, and the difference was statistically significant (*P* < .05). The MLR of PBC was also higher than that of HCs (*P* < .01) (Figure 1). The RLR (Figure 2) of PBC was significantly higher than that of AIH and HCs (*P* < .01).

### Receiver Operating Characteristic ***Curve Analysis Results***

**Figure 3.**
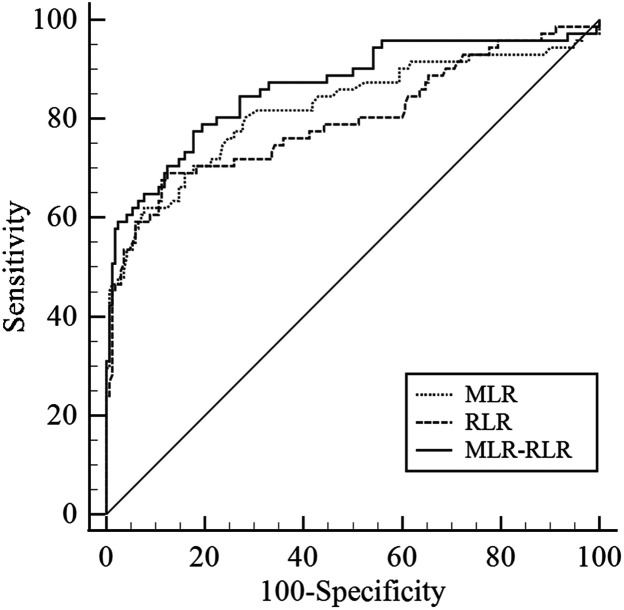
ROC curve for the evaluation value of PBC by MLR, RLR, and MLR-RLR. ROC, Receiver operating characteristic curve; PBC, primary biliary cholangitis; MLR, monocyte-to-lymphocyte ratio; RLR, red cell distribution width-to-lymphocyte ratio.

**Figure 4.**
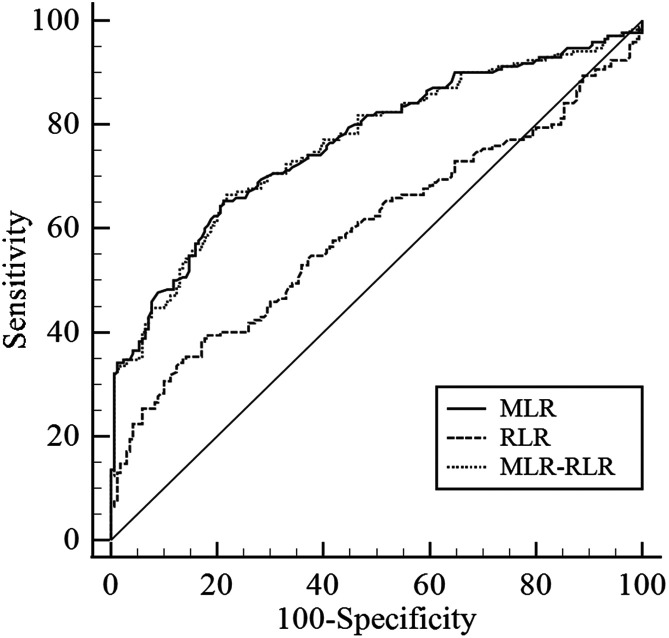
ROC curve for the evaluation value of autoimmune hepatitis by MLR, RLR, and MLR-RLR. ROC, receiver operating characteristic curve; MLR, monocyte-to-lymphocyte ratio; RLR, red cell distribution width-to-lymphocyte ratio.

Figure 3 shows that ROC curve for the evaluation value of PBC by MLR, RLR, and MLR-RLR. The optimal MLR cut-off value of 0.278 had 62% sensitivity and 92% specificity (AUC = 0.821, 95% CI, 0.766-0.867, *P* < .01). The optimal RLR cut-off value of 0.101 had 69% sensitivity and 88% specificity (AUC = 0.797, 95% CI, 0.741-0.846, *P* < .01). The optimal MLR-RLR cut-off value of 0.280 had 75% sensitivity and 85% specificity (AUC = 0.868, 95% CI, 0.818–0.908, *P* < .01). Figure 4 shows that ROC curve for the evaluation value of AIH by MLR, RLR, and MLR-RLR. The optimal MLR cut-off value of 0.232 had 65% sensitivity and 79% specificity (AUC = 0.764, 95% CI, 0.716–0.808, *P* < .01). The optimal RLR cut-off value of 0.092 had 38% sensitivity and 83% specificity (AUC = 0.596, 95% CI, 0.542-0.648, *P* < .01). The optimal MLR-RLR cut-off value of 0.514 had 64% sensitivity and 80% specificity (AUC = 0.762, 95% CI, 0.714–0.807, *P* < .01).

### Correlations of Monocyte-to-Lymphocyte Ratio and Red Cell Distribution Width-to-Lymphocyte Ratio with the Clinical Characteristics of Primary Biliary Cholangitis Patients

**Figure 5.**
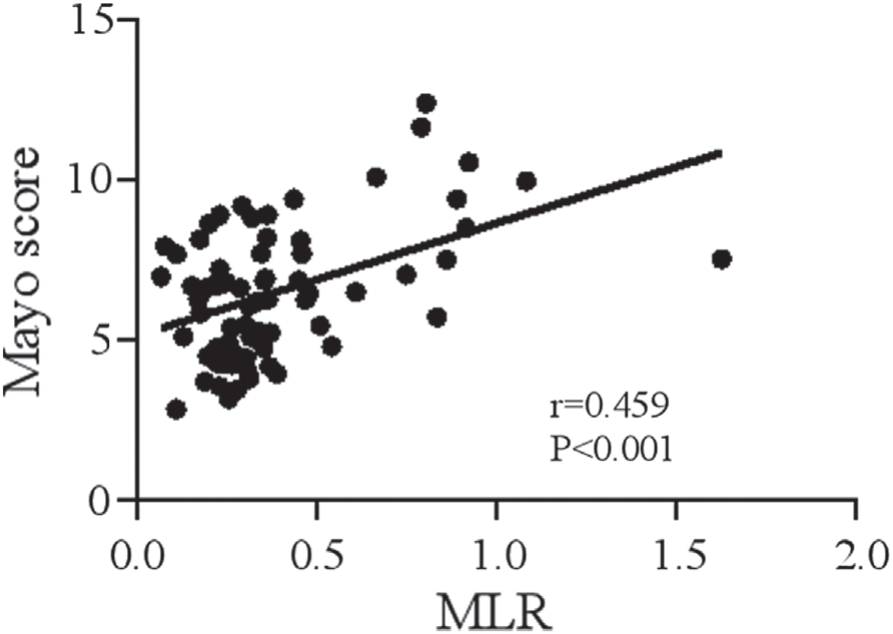
Correlation of MLR with Mayo score in PBC. PBC, primary biliary cholangitis; MLR, monocyte-to-lymphocyte ratio. RLR, red cell distribution width-to-lymphocyte ratio; PBC, primary biliary cholangitis.

**Figure 6.**
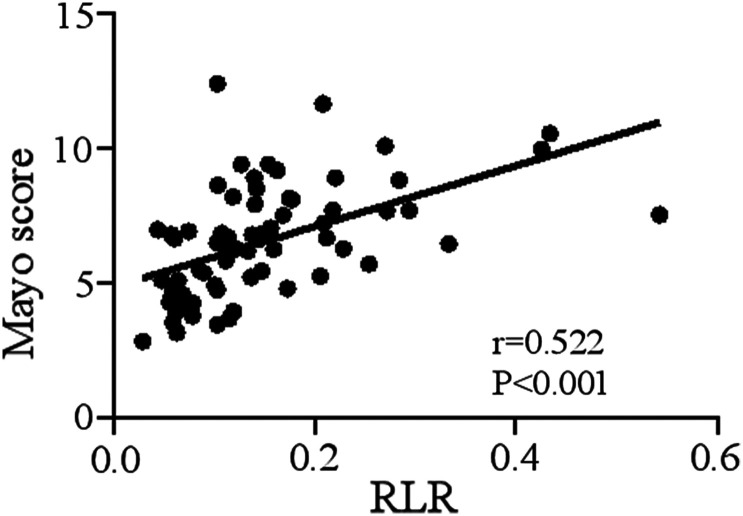
Correlation of RLR with Mayo score in PBC. RLR, red cell distribution width-to-lymphocyte ratio; PBC, primary biliary cholangitis.

Figure 5 shows that MLR was positively correlated with the Mayo score (*r* = 0.459, *P* < .001). Figure 6 shows that RLR was positively correlated with the Mayo score (*r* = 0.522, *P* <.001). In PBC patients, RLR was negatively correlated with C3 (*r* = -0.384, *P* = .008) and C4 (*r* = -.321, *P* = 0.028). There was no correlation with other indexes in PBC patients (see Table 2). Further, multiple linear regression was utilized to analyze the relationship between MLR, RLR, IgA, IgG, age, sex, and Mayo score. After adjusting for other factors, RLR was the main factor affecting Mayo score (Table 3).

### The Relationships Between Monocyte-to-Lymphocyte Ratio, Red Cell Distribution Width-to-Lymphocyte Ratio, and Child–Pugh Classification in Primary Biliary Cholangitis Patients

**Figure 7.**
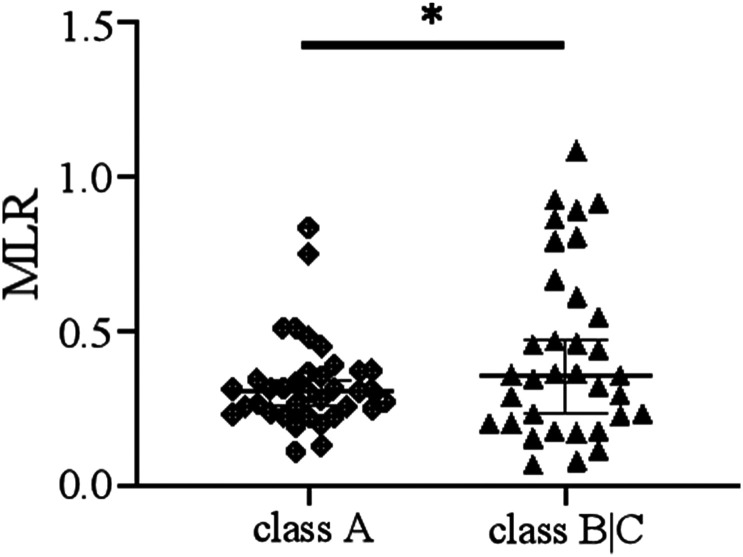
The relationship between MLR and Child–Pugh classification of PBC. *P < .05, **P < .01. MLR, monocyte-to-lymphocyte ratio; PBC, primary biliary cholangitis.

**Figure 8.**
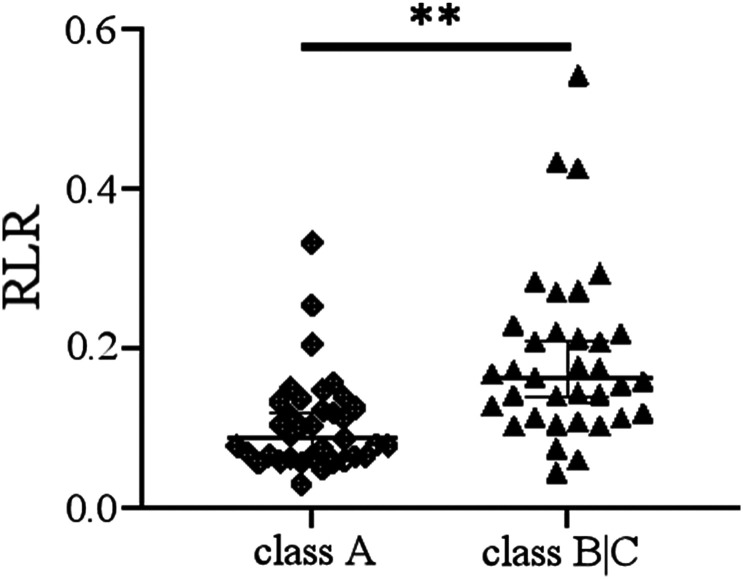
The relationship between RLR and Child–Pugh classification of PBC. *P < .05, **P < .01. RLR, red cell distribution width-to-lymphocyte ratio; PBC, primary biliary cholangitis.

In Child–Pugh classification, the MLR (Figure 7) in class B|C was 0.36 (0.20, 0.67), which was significantly higher than the MLR of class A, 0.31 (0.24, 0.37), *P* < .05. The RLR (Figure 8) in class B|C was 0.16 (0.11, 0.22), which was significantly higher than the RLR of class A, 0.09 (0.06, 0.13), *P* < .01.

## Discussion

Primary biliary cholangitis is a chronic intrahepatic cholestatic disease characterized by nonsuppurative destructive cholangitis.^18^ Currently, a study has shown that inflammation is one of the main pathophysiological mechanisms for the development and progression of PBC.^19^ Monocyte-to-lymphocyte ratio and RLR, as novel markers of inflammation, have been studied in various inflammatory diseases. For instance, septicemia and community-acquired pneumonia are associated with elevated MLR levels.^20,21^ Increased RLR has been reported to be associated with the severity of cirrhosis.^13^

Our results show that MLR and RLR were significantly increased in PBC patients, and MLR and RLR were positively correlated with the Mayo score, suggesting that the prognosis of PBC patients was worse with the increase of MLR and RLR. Besides, MLR and RLR were higher in class B|C than in class A of the Child–Pugh classification, suggesting that elevated MLR and RLR were associated with poor hepatic functional reserve.

Monocytes and lymphocytes in blood routine are part of the immune system and are important indicators for evaluating inflammatory response. Our study found that MLR was significantly increased in PBC patients, which was thought to be related to the increase of monocytes and decrease of lymphocytes in peripheral blood of PBC patients. Peng et al^22^ found that elevated CD14^low^ CD16^+^ monocyte subsets in PBC promote liver injury and inflammatory response. Another study confirmed that the PBC liver showed monocyte infiltration in portal tracts, especially around the bile ducts.^23^ Primary biliary cholangitis is an autoimmune disease in which lymphocytes play a very important role in immune surveillance. The decrease of lymphocyte count may be linked to the apoptosis and dysfunction of immune cells. Previous studies have reported that the progression of advanced cirrhosis is related to the gradual decline of the lymphocyte count.^24^ Monocyte-to-lymphocyte ratio is less affected by other factors such as infection, drugs, and physical conditions than the absolute number of individual cell types. To sum up, the predictive efficacy of MLR is more objective than either parameter alone.

Red cell distribution width is a parameter of cell heterogeneity, and its increase is related to the immune status of the body. Wu et al^25^ indicated that RDW increased with the deterioration of liver function and was positively associated with the incidence of liver failure. Red cell distribution width-to-lymphocyte ratio was calculated as the ratio of RDW to lymphocytes. Red cell distribution width-to-lymphocyte ratio is considered an indicator of inflammation. We found that RLR was negatively correlated with C3 and C4. In addition, after adjusting for IgA, IgG, age, and sex, it was found that RLR was independently associated with Mayo score, indicating that RLR can predict the prognosis of PBC patients to some extent. A recent study has shown that RLR can be used as a biomarker to predict the prognosis of patients with renal cell carcinoma, improving the predictability of survival.^26^

The most important finding of our study is that MLR and RLR have good diagnostic value for patients with early PBC. The combination of MLR and RLR produced an AUC of 0.868, which is higher than the predicted value of any single indicator. Combined, then, these 2 indicators have the potential to be used as a hematology marker in the diagnosis of PBC.

There are a few limitations in this study. First, our study was a retrospective design. Therefore, these conclusions need to be further verified by prospective studies. Second, the relatively small sample size may limit our further findings in patients with PBC. Finally, we did not explore how treatment affected MLR and RLR in PBC patients. Despite these limitations, we found that MLR and RLR were significantly associated with inflammatory markers and disease prognosis in PBC patients.

In conclusion, elevated levels of MLR and RLR may prove to be useful markers for estimating the prognosis of PBC, and combined detection of MLR and RLR has clinical diagnostic value in patients with PBC.

## Figures and Tables

**Table 1.** Characteristics of Subjects

	PBC (n = 71)	AIH (n = 170)	HCs (n = 170)	F/χ^2^	*P*
Age (years)	58 (54, 68)	58 (51, 66)	57 (55, 61)	0.857	.425
Gender (male/female)	4/67 (5.97%)	26/144 (18.06%)	20/150 (13.33%)	4.418	.166
WBC (×10^9^/L)	5.34 (4.10, 7.39)	6.72 (4.90, 8.07)	5.80 (4.96, 6.81)	9.853	<.001
Hemoglobin (g/L)	106.70 (91.40, 118.00)	123.20 (112.93, 133.78)	133.15 (126.00, 139.83)	93.589	<.001
Platelet (×10^9^/L)	161.00 (96.40 , 248.80)	202.7 (149.75, 260.5)	259.65 (213.80, 301.60)	34.504	<.001
Neutrophil (×10^9^/L)	2.92 (2.47, 4.25)	3.56 (2.46, 5.05)	3.12 (2.72, 3.38)	9.212	<.001
Lymphocyte (×10^9^/L)	1.28 (0.96, 2.32)	1.81 (1.45, 2.46)	2.01 (1.62, 2.50)	16.905	<.001
Monocyte (×10^9^/L)	0.48 (0.33, 0.56)	0.50 (0.39, 0.70)	0.37 (0.32, 0.45)	28.696	<.001
RDW (%)	16 (14, 18)	15 (14, 16)	14 (13, 14)	27.541	<.001
MLR	0.31 (0.23, 0.46)	0.27 (0.21, 0.36)	0.19 (0.15, 0.23)	48.094	<.001
RLR	0.12 (0.08, 0.17)	0.08 (0.06, 0.11)	0.07 (0.06, 0.09)	47.480	<.001
GGT (U/L)	304 (97, 558)	279 (115.73, 552.75)	26 (17, 35.70)	77.500	<.001
ALT (U/L)	67 (36, 118)	93 (60, 172.25)	18 (14, 25.25)	46.880	<.001
AST (U/L)	97 (63, 135)	102 (74, 158.25)	20 (17, 25)	55.066	<.001
ALP (U/L)	287 (189, 467)	207 (120.25, 421.25)	75 (65, 90)	92.228	<.001

Parameters were expressed with median (25th, 75th percentiles).

WBC, white blood cell count; RDW, red cell distribution width; MLR, monocyte-to-lymphocyte ratio; RLR, red cell width distribution-to-lymphocyte ratio; GGT, gamma glutamyl transferase; ALT, alanine aminotransferase; AST, aspartate aminotransferase; ALP, alkaline phosphatase.

**Table 2.** Correlations of MLR and RLR with Clinical Indicators in PBC Patients

	MLR		RLR	
	*r*	*P*	*r*	*P*
C3 (g/L)	0.026	.864	−0.384	.008
C4 (g/L)	−0.007	.964	−0.321	.028
IgA (g/L)	0.112	.392	0.191	.144
IgG (g/L)	0.200	.126	0.182	.165
IgM (g/L)	−0.193	.140	−0.185	.156
ESR (mm/h)	0.017	.907	0.007	.964

MLR, monocyte-to-lymphocyte ratio; RLR, red cell distribution width-to-lymphocyte ratio; C3, complement 3; C4, complement 4; IgA, immunoglobulin A; IgG, immunoglobulin G; IgM, immunoglobulin M; ESR: erythrocyte sedimentation rate.

**Table 3.** The Multiple Linear Analysis of the Correlation between MLR, RLR, and Mayo Score

	B	Std. Error	95% CI	*P*
Constant	1.831	1.170	−0.518 to 4.179	.124
MLR	1.049	1.060	−1.079 to3.178	.327
RLR	6.359	2.950	0.436 to12.281	.036
IgA (g/L)	0.537	0.141	0.254 to0.821	<.001
IgG (g/L)	−0.005	0.045	−0.096 to0.085	.905
Age (years)	0.034	0.018	−0.002 to0.069	.063
Gender	−0.522	0.850	−2.229 to 1.185	.542

MLR, monocyte-to-lymphocyte ratio; RLR, red cell distribution width-to-lymphocyte ratio; IgA, immunoglobulin A; IgG, immunoglobulin G.
